# New Perspectives in Phlebology and Lymphology

**DOI:** 10.3390/jcm11071902

**Published:** 2022-03-29

**Authors:** Attilio Cavezzi

**Affiliations:** Eurocenter Venalinfa, 63074 San Benedetto del Tronto (AP), Italy; info@cavezzi.it

## 1. Introduction

The recent research on chronic degenerative diseases (CDD), such as obesity, diabetes, neurodegeneration, atherosclerosis, autoimmune diseases, cancer and aging itself, has shown that Venous and Lymphatic Diseases (VLD) may play an important role in their development, de facto pushing phlebology and especially lymphology under a spotlight in biomedical research. Veins and especially lymphatics have a determinant role in the tissue homeodynamics/clearance, and similarly, it is known that tissue degeneration accompanies all CDD (and VLD); consequently, venous and lymphatic morpho-dynamic abnormalities may represent important components in the complex puzzle of CDD pathophysiology.

The clinical manifestations of VLD also reflect several other epigenetics-related co-causal factors (such as obesity, erroneous lifestyle, drug intake, postural changes, musculo-vascular pump dysfunction, etc.) which certainly interfere with the onset, progression and therapeutic response of these diseases [1].

The correlation between CDD and VLD causes us to consider phlebolymphology not just as a branch of the traditional western-reductionist biomedical science, but as an integrated discipline with a keen interest in using the approaches of translational medicine and of epigenetics in the overall management of patients affected by VLD [1]—a trend which is influencing all biomedical science.

These new trends are now being accompanied by a significant reappraisal of diagnostic and therapeutic pathways for VLD: the possibility to exploit pharmacology and technology has sparked innovation in diagnostics and treatments for varicose vein disease, deep and superficial vein thrombosis, post-thrombotic syndrome (PTS), as well as for leg venous ulcers (LVU), pelvic vein insufficiency (PVI) and lymphedema with its complications. Similarly, the adherence to the principles of cost-effectiveness and appropriateness of the available diagnostic/therapeutic means—a basic requirement in medical practice—should keep phlebolymphology from degenerating into a money-making/money-eating machine and push it to engage in virtuous propositions of a “wiser” approach that takes into account the basic pathophysiology mechanisms of each disease as well [1,2].

In fact, beyond all evidence-based decisional pathways, a newer tendency towards a more comprehensive and cost–benefit-based and patient-centred approach, the so-called “choosing wisely” movement, is permeating the medical world [1,3]; this tendency undoubtedly matches the need for a more reasoned allocation of resources, even more in these complex times which impose a reappraisal of healthcare spending.

## 2. Phlebology

Varicose veins (VV) represent the most frequent chronic venous disease in the general population, reaching a 21% pooled prevalence [4], and although innovative treatments are continuously proposed, ineluctably, a 30% or higher mid/long-term recurrence rate is documented by most publications [1,2,4]. The most recent endovenous procedures, based for example on laser, radiofrequency, (catheter-)foam sclerotherapy, glue and mechanochemical ablation, are showing promising results, but a higher degree of evidence is still required as to their long-term efficacy [1,2,4,5]. Surgery is still considered a feasible and useful therapeutic procedure for VV, but only if practised in a mini-invasive and tailored modality in order to improve its efficacy, safety and cost-effectiveness [1,4,6].

However, regardless of the therapeutic options, a thorough colour-duplex ultrasound (CDU)-based diagnostic approach to the varicose patient is fundamental to tailor a specific treatment and improve its technical and strategic elements. As PVI and abdominal vein obstruction may relevantly impact the treatment of VV disease and its recurrence rate [1,4,7], greater attention should be paid to this peculiar morpho-hemodynamic condition through the combination of CDU with other radiological diagnostic tools, such as computed tomography/magnetic resonance-based venography. Actually, in the presence of lower limb VV related also to abdomino-pelvic vein insufficiency, this combined approach may offer some advantages [8].

In fact, a high incidence of PVI and pelvic refluxes has been documented in varicose patients, especially in the case of non-saphenous varices and in pluriparous women [4,8,9]. In most cases, refluxes in gonadal veins are elicited and a sclero-embolization procedure may be indicated in these patients, especially when frankly symptomatic [1,4,8,9].

The treatment of minor varicosities, such as reticular varices and telangiectasias, relies on sclerotherapy which has undoubtedly proven its value through past and recent literature data. The need for a detailed CDU evaluation before any treatment of these C1 (CEAP clinical classification) cases has been repeatedly highlighted [4] in order to avoid those side effects, complications and poor outcomes attributable to a wrong strategy. In fact, saphenous incompetence and/or truncular vein refluxes are often associated with apparently isolated minor varicosities [4], and these major refluxes should be addressed firstly, before any treatment on spider veins.

Regardless of the undisputed role of liquid (preferably) or foam sclerotherapy in these patients affected by minor varicosities, a number of alternative techniques have been proposed in recent years; however, these innovative technology-based interventions (e.g., laser, cryo-laser, cryo-sclerotherapy, ultrasounds) are burdened with higher costs and technical complexities. Furthermore, the outcomes of these methods seem to be similar, if not inferior, to those of the inexpensive and feasible sclerotherapy-based management [1,4], which should induce to a better reasoned therapeutic approach to these venous disorders.

Undoubtedly, the strong pressure of industry players and of (often excessively demanding) patients in search of innovative and appealing technologies represents an issue for all those physicians treating reticular varices and telangiectasias which pertain to cosmetic medicine as well. Cost-effectiveness criteria certainly favour the use of sclerotherapy in the vast majority of cases, hence non-medical reasons should hopefully have a limited space in the decisional therapeutic process.

In VV treatment, the topic of thromboprophylaxis represents an important and debated issue in light of the need to minimise possible post-operative complications. The currently employed pharmaceutical (e.g., low molecular weight heparins) and mechanical methods (e.g., medical compression stockings (MCS) have been repeatedly tested with contrasting outcomes. Despite these uncertainties, experts have agreed on the benefit of the post-operative short-term use of MCS or bandages with references to side effects [4,10]. Interestingly, for innovative and less invasive techniques such as endovenous ablation by means of long-wave double-ring laser, post-operative compression stockings may show a beneficial role on a few symptoms (e.g., pain sensation); however, this positive effect is shown to be significant only if phlebectomies or sclerotherapy are also performed on the varicose tributaries [11].

Overall, to choose the proper prophylactic method and its duration, an adequate individual risk-stratification assessment is advocated: a recent prospective observational study addressed this issue and showed that both the Caprini score and venous clinical severity score questionnaire may be useful tools to stratify the risk of post-operative deep venous thrombosis (DVT) [12].

Another extremely debated topic regards DVT and PTS, where doubtful evidence still exists on the utility, kind and duration of the compression device and regime [1,4,10,13]. In fact, after the controversial SOX trial [14], which questioned the usefulness of MCS at reducing the incidence of PTS following an acute DVT, a few well-conducted studies and one Cochrane review [15,16,17] have been published on this topic. Overall, they re-confirmed the value of compression garments to reduce PTS in limbs affected by DVT, but likely on an individualised basis. In any case, it has been highlighted that more high-quality evidence is needed to definitely clarify this indication in view of the heterogeneity of the available data.

Conversely, the beneficial role of compression therapy is being increasingly confirmed in LVU, where ambulatory venous hypertension and microcirculation/tissue derangements reach the worst levels: in these contexts, besides the expensive and partially validated innovative techniques based on skin grafting or advanced medications and local dressings, compression therapy exerts an undisputed and fundamental role in the increase of the interstitial pressure, reduction of microcirculatory stasis, and hence on the healing process of phlebostatic ulcers [1,4,10]. Yet, the quite high recurrence rate of this late stage of chronic venous insufficiency imposes a reappraisal of its management; in fact, medical practice should acknowledge and address the great importance of other relevant factors (e.g., musculo-vascular pump dysfunction, obesity, deleterious drug intake) which deteriorate microcirculation and tissue balance. Similarly, there is growing evidence about the appropriateness of an intervention on saphenous incompetence in many patients affected by these highly prevalent (1–3%) [4] skin dystrophies. Because of the LVU-associated relevant socio-economic burden, several multi-modality diagnostic and therapeutic approaches are being proposed to achieve a cost-saving and effective multidisciplinary management of these patients.

When LVU are associated with diabetes and/or to peripheral arterial obliterating disease, a more complicated course of the lesions is expected; thus, a careful diagnostic procedure and treatment is advocated in these mixed arterio-venous-neuropathic ulcers. More recently, literature data have pointed out how the use of compression devices may be indicated and beneficial also in these specific cases, provided that the limb has an ankle-brachial index above 0.5 [18].

The management of deep vein occlusion (DVT) or chronic obstruction (SPT) equally represents a debated topic, with conservative treatment being much more practised over any interventional approach. In fact, the costly exploitation of advanced technologies, such as intravenous ultrasound, pharmaco-mechanical thrombolysis, stents, ultrasound-based thrombolysis or complex surgical options have been considered as appropriate only in selective cases, such as ilio-caval thromboses and late and therapy-resistant stages of chronic venous insufficiency [1,4].

## 3. Lymphology

When treating lymphedema, compression therapy represents the cornerstone modality within the combined decongestive approach in acute and intensive care as well as in the maintenance phase [19,20]. Regrettably, through physical and rehabilitative treatment, suboptimal long-term outcomes are generally achieved for this chronic disabling disease which requires lifelong care and an adequate patient compliance [20]. The objective need for better outcomes and for this lifelong therapy in patients affected by lymphedema is pushing both medical professionals and industries to research new solutions for diagnostic and therapeutic purposes.

Besides the innovative exploitation of a few diagnostic methods, such as bioimpedance spectroscopy and innovative lymphatic imaging technologies, several therapeutic proposals are being proposed to improve lymph stasis.

Together with new drugs and nutraceuticals, measures to improve parasympathetic systems, gene treatments and stem cells—just to name a few—have entered the future research and clinical practice of lymphedema management, with a view of an interdisciplinary medical pathway [1,21,22].

By virtue of the necessity of a higher degree of self-management of lymphedema and of other VLD, adjustable compression wraps have been proposed as a possible tool in this regard. The efficacy and safety of this compression device has been demonstrated through a few studies in recent decades, both in lymphedema and in VLU [1,4,10,19,20].

Furthermore, in lymphedema patients excisional or reconstructive surgical treatments may be selectively indicated, especially where conservative treatment has had little if no efficacy [1,20]. Lymphostatic verrucosa is one of the late complications of lymphedema and it does represent a specific issue for the conventional combined decongestive therapy; a role for shaving surgery in this condition has been highlighted in past and recent publications, with reported beneficial mid-term outcomes [23].

Overall, the conservative physical-rehabilitative approach for lymphedema, based on manual lymphatic drainage, compression devices, electric medical equipment, skin care, drugs and nutraceuticals, may provide remarkable short-term benefits. Conversely, mid- to long-term outcomes of this decongestive therapy often do not match physicians’ and patients’ expectations, due to the complexity of the disease itself and to the interplay of subjective and environmental elements in lymphedema prognosis [20].

However, as with most chronic venous diseases, the course of lymphedema and the response to any treatment are strongly influenced by several non-vascular factors which impact lymph stasis and limb oedema more generally. Among these, diabesity (diabetes and obesity syndrome) stands out as a major issue of concern. In fact, this metabolic syndrome is spreading as a true pandemic worldwide, and it was repeatedly shown to dramatically worsen most VLD, lymphedema and VLU primarily [1].

Taking these aspects into consideration, an integrated medical approach to lymph stasis, based also on translational medicine, has been proposed [1,21]: improper nutrition and lifestyle, stress and an altered autonomic nervous system, and detrimental drugs may relevantly impact lymphedematous patients, who may consequently benefit from an improvement of several life-lasting erroneous habits.

## 4. Conclusions

Overall, new perspectives are opening on the diagnosis and therapy of VLD following the progress of biomedical science. A wise, ethical and sustainable exploitation of the innovative pharmaceutical and technological advancements could certainly lead to a safer and more effective medical management of these diseases. Similarly, and more importantly, the inclusion of epigenetic science and translational medicine, with a higher attention to the patient’s lifestyle, nutrition, environmental and psycho-social aspects, could contribute to overcoming the frequently reductionist approach of current medical practice in general.
